# Crystal structure of 2-(benzo[*d*]thia­zol-2-yl)-3,3-bis­(ethyl­sulfan­yl)acrylo­nitrile

**DOI:** 10.1107/S2056989022002572

**Published:** 2022-03-10

**Authors:** Rasha A. Azzam, Galal H. Elgemeie, Rasha E. Elsayed, Nagwa M. Gad, Peter G. Jones

**Affiliations:** a Chemistry Department, Faculty of Science, Helwan University, Cairo, Egypt; b Institut für Anorganische und Analytische Chemie, Technische Universität Braunschweig, Hagenring 30, D-38106 Braunschweig, Germany

**Keywords:** benzo­thia­zol, acrylo­nitrile, crystal structure

## Abstract

In the title compound, C_14_H_14_N_2_S_3_, the double-bond system of the acrylo­nitrile moiety is significantly non-planar, with absolute *cis* torsion angles of 13.9 (2) and 15.1 (2)°. The ring system and the double bond system subtend an inter­planar angle of 11.16 (4)°. The wide angle C—C(CN)=C of 129.40 (12)° may be associated with a balance between planarity and avoidance of a very short S⋯S contact.

## Abstract 2

The double-bond system of the acrylo­nitrile moiety is significantly non-planar and displays one very wide angle C—C(CN)=C.

## Chemical context

Research into medicinal chemistry based on benzo­thia­zoles has become a fast developing and progressively more active topic. The high degree of structural diversity has proved to be important in the search for new effective treatments (Ammazzalorso *et al.*, 2020 ▸; Elgemeie, 1989 ▸). A large number of therapeutic agents based on benzo­thia­zole systems have been synthesized and evaluated in terms of their pharmacological properties (Gill *et al.*, 2015 ▸; Fathy *et al.*, 1988 ▸). Much information about benzo­thia­zoles has been reported in the scientific literature, describing their anti-inflammatory, anti­microbial, neuroprotective, anti­convulsant and anti­proliferative effects (Seenaiah *et al.*, 2014 ▸). The mol­ecular mechanisms responsible for this variety of pharmacological activity have not been completely established, and various biological pathways have been indicated as possible targets of this class of mol­ecules (Keri *et al.*, 2015 ▸). We are engaged in developing synthetic strategies for benzothaizole systems that show important biological activity as novel anti­microbial and anti­viral agents (Azzam *et al.* 2017*a* ▸,*b* ▸, 2020*a* ▸,*b* ▸,*c* ▸, 2021 ▸; Elgemeie *et al.*, 2000*a* ▸,*b* ▸; 2020 ▸).

As an extension of this research (Fathy & Elgemeie, 1988 ▸; Elgemeie & Elghandour, 1990 ▸), we report here a novel benzo­thia­zole cyano­ketene di­thio­acetal (**2**). Compound **2** was synthesized by the reaction of 2-cyano­methyl­benzo­thia­zole **1** with carbon di­sulfide in the presence of sodium ethoxide, followed by alkyl­ation with ethyl iodide. The structure of **2** was originally based on its elemental analysis and spectroscopic data (see *Experimental*). In order to establish the structure of the compound unambiguously, the crystal structure was determined.

## Structural commentary

**Figure 1:**
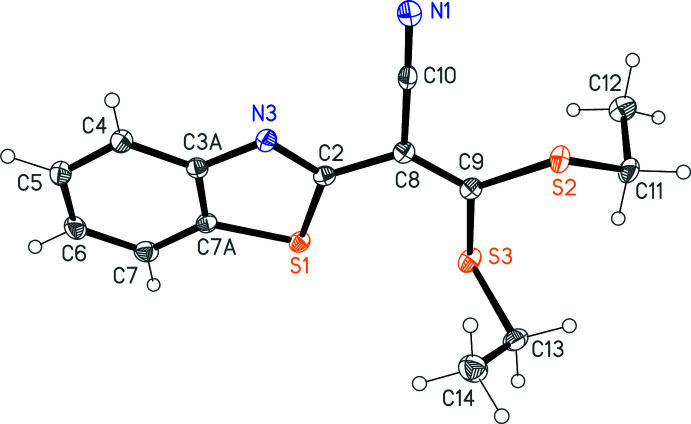
The mol­ecule of **2** in the crystal. Ellipsoids represent 50% probability levels.

The mol­ecule of **2** is shown in Fig. 1 ▸. The heterocyclic system is coplanar to within an r.m.s. deviation of only 0.007 Å, and its dimensions are as expected (a selection of mol­ecular dimensions are presented in Table 1 ▸). There is appreciable twisting of *ca* 14° about the double bond C8=C9 (see torsion angles in Table 1 ▸), so that the ‘plane’ of the atoms C2, C8, C9, C10, S2 and S3 displays an r.m.s. deviation of 0.14 Å; the two planes subtend an inter­planar angle of 11.16 (4)°. The angle C2—C8=C9 (formally *sp* ^2^) is strikingly wide, at 129.40 (12)°; for comparison, the corresponding angles in the five structures mentioned below (with refcodes) range from 122–126°. One might speculate that this large angle and the deviation from planarity about the double bond represent aspects of a compromise between (i) achieving coplanarity of the heterocycle with the double-bond system and (ii) avoiding too short an S⋯S contact. The intra­molecular S⋯S distances are S1⋯S3 = 3.1155 (5) and S2⋯S3 = 3.0496 (5) Å. The ethyl groups project to opposite sides of the mol­ecule. 
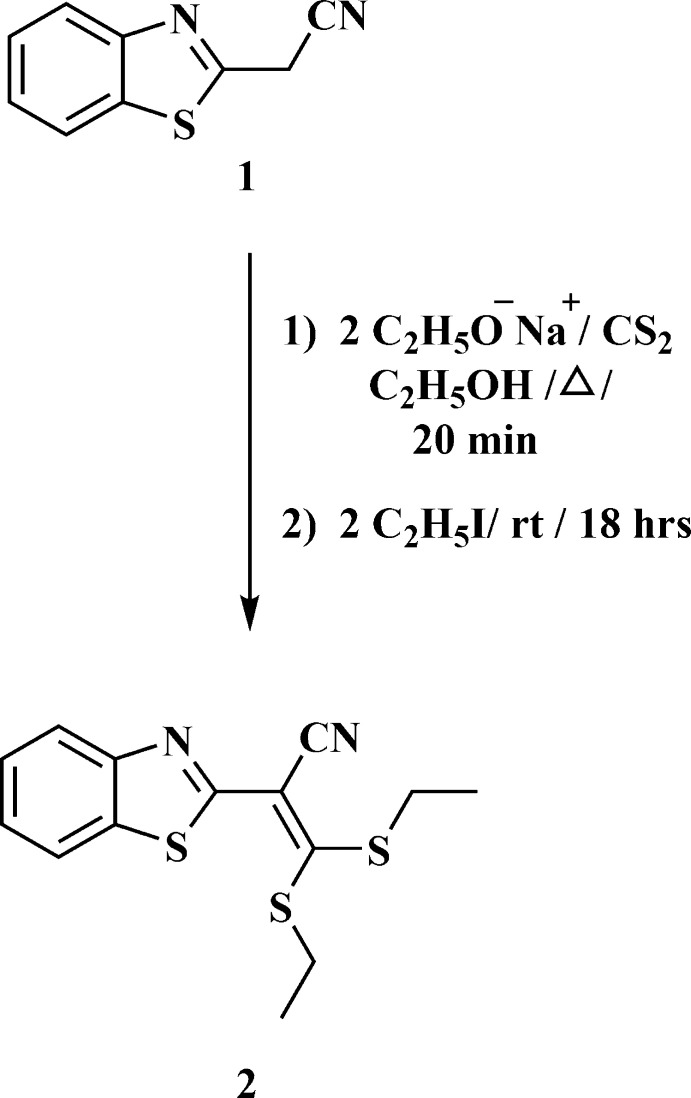


## Supra­molecular features

**Figure 2:**
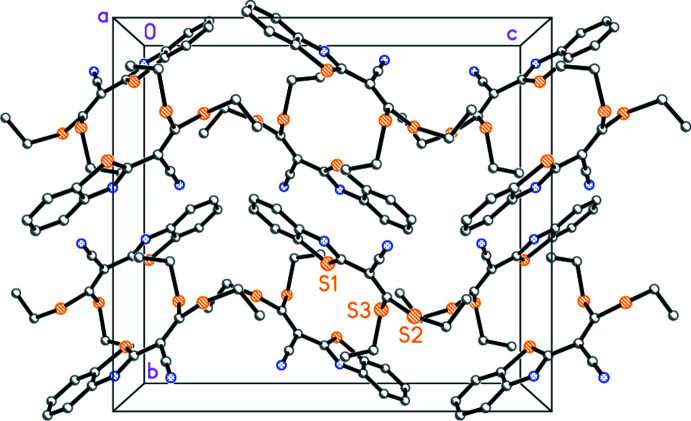
Crystal packing of **2** viewed parallel to the *a* axis (hydrogen atoms omitted for clarity). The loose association of the heterocyclic systems across inversion centres can be recognized in the central horizontal rows of rings.

The mol­ecular packing is fairly featureless; a general view is given in Fig. 2 ▸ and some borderline possible ‘weak’ hydrogen bonds are listed in Table 2 ▸. The main feature is the loose association of pairs of mol­ecules across inversion centres, whereby the heterocyclic systems face each other; however, there is a considerable offset. The centroids of the five-membered rings lie 3.72 Å apart, and the shortest contact is C7*A*⋯C7*A*′ (operator 1 − *x*, 1 − *y*, 1 − *z*) 3.741 (2) Å. The sulfur atom S1 lies 3.61 Å from the centroid of the six-membered ring in the facing mol­ecule; such potential S⋯π inter­actions have been discussed by *e.g*. Ringer *et al.* (2007 ▸) and Silva *et al.* (2018 ▸).

## Database survey

Searches of the Cambridge Structural Database (Groom *et al.*, 2016 ▸) were performed using *ConQuest* Version 2021.3.0. A search for the moiety benzo[*d*]thia­zol-2-yl joined to C(CN)=C gave 27 hits, but none in which any further atom at the double bond was sulfur. A search for the group C—C(CN)=C(S—C)_2_, with the first carbon atom three-coordinate, both sulfur atoms two-coordinate and not involving cyclicity, gave only five hits. The refcodes, references and absolute *cis* torsion angles NC—C=C—S were as follows: CIYDIY, Kumar *et al.* (2008 ▸), 9.9°; MTBCEY, Abrahamsson *et al.* (1974 ▸), 15.4°; VAPJAA, Azzam *et al.* (2017*c* ▸), 7.3°; VELSIP, Peng *et al.* (2006 ▸), 3.6°; ZEDJEX, Osaka *et al.* (1994 ▸), 10.5°.

## Synthesis and crystallization

A mixture of sodium ethoxide (0.08 mol) and 2-cyano­methyl­benzo­thia­zole (0.04 mol) in absolute ethanol (100 ml) was refluxed for 20 min. After cooling, carbon di­sulfide (0.04 mol) was added gradually and then the solution was warmed for 20 min. Ethyl iodide (0.08 mol) was then added, and the reaction mixture was stirred overnight at room temperature. The solution was poured onto ice–water and the solid product thus formed was filtered off. The product was purified by dissolving it in hot petroleum ether, filtering, and allowing the solution to cool. The solid that formed was recrystallized from DMF to give pale-yellow crystals, m.p. = 366–368 K, yield 72%; IR (KBr, cm^−1^): *υ* 3056 (ArCH), 2924 (CH_3_), 2213 (CN), 1502 (C=N); ^1^H NMR (300 MHz, DMSO-*d_6_*): *δ* 1.27–1.34 (*m*, 6H, 2 SCH_2_CH_3_), 3.16–3.23 (*m*, 4H, 2 SCH_2_CH_3_), 7.50–7.57 (*m*, 2H, benzo­thia­zole H), 8.04–8.15 (*m*, 2H, benzo­thia­zole H); analysis, calculated for C_14_H_14_N_2_S_3_ (306.47): C% 54.87; H% 4.60; N% 9.14; S% 31.39; found: C% 54.85, H% 4.58; N% 9.16; MS *m*/*z* (%): 306 (*M* ^+^, 15%), 276 (100%), 273 (57%), 248 (26%), 217 (76%), 204 (26%), 146 (20%).

## Refinement

Crystal data, data collection and structure refinement details are summarized in Table 3 ▸. The methyl groups were refined as idealized rigid groups allowed to rotate but not tip, with C—H = 0.98 Å and H—C—H = 109.5°. Other hydrogen atoms were included using a riding model starting from calculated positions (C—H_aromatic_ = 0.95, C—H_methyl­ene_ = 0.99 Å). The *U*(H) values were fixed at 1.5 or 1.2 times the equivalent *U*_iso_ value of the parent carbon atoms for methyl and non-methyl hydrogen atoms, respectively.

## Supplementary Material

Crystal structure: contains datablock(s) I, global. DOI: 10.1107/S2056989022002572/ex2055sup1.cif

Structure factors: contains datablock(s) I. DOI: 10.1107/S2056989022002572/ex2055Isup2.hkl

Supporting information file. DOI: 10.1107/S2056989022002572/ex2055Isup3.cml

CCDC reference: 2156777

Additional supporting information:  crystallographic information; 3D view; checkCIF report

## Figures and Tables

**Table 1:** Selected geometric parameters (Å, °)

S1—C7*A*	1.7371 (13)	C3*A*—C7*A*	1.4057 (17)
S1—C2	1.7519 (13)	C9—S3	1.7489 (13)
C2—N3	1.3078 (16)	C9—S2	1.7526 (13)
N3—C3*A*	1.3813 (16)		
			
C7*A*—S1—C2	88.97 (6)	C9—C8—C2	129.40 (12)
N3—C2—S1	115.52 (9)	C10—C8—C2	111.90 (10)
C2—N3—C3*A*	110.98 (11)	C8—C9—S3	121.13 (10)
N3—C3*A*—C7*A*	115.03 (11)	C8—C9—S2	117.68 (10)
C3*A*—C7*A*—S1	109.49 (9)	S3—C9—S2	121.14 (7)
C9—C8—C10	118.69 (11)		
			
C2—C8—C9—S3	13.90 (19)	C2—C8—C9—S2	−163.46 (10)
C10—C8—C9—S2	15.10 (16)	C8—C9—S2—C11	−146.43 (10)

**Table 2:** Hydrogen-bond geometry (Å, °)

*D*—H⋯*A*	*D*—H	H⋯*A*	*D*⋯*A*	*D*—H⋯*A*
C7—H7⋯S2^i^	0.95	3.02	3.6083 (13)	122
C12—H12*B*⋯S1^ii^	0.98	3.03	3.9677 (15)	161
C13—H13*A*⋯N3^i^	0.99	2.68	3.5746 (17)	151
C14—H14*A*⋯S1^iii^	0.98	2.91	3.7648 (15)	146
C14—H14*B*⋯N3^iv^	0.98	2.63	3.5277 (18)	152

Symmetry codes: (i) 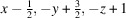 ; (ii) 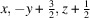 ; (iii) 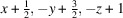 ; (iv) 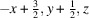 .

**Table 3:** Experimental details

Crystal data	
Chemical formula	C_14_H_14_N_2_S_3_
*M*_r_	306.45
Crystal system, space group	Orthorhombic, *P* *b* *c* *a*
Temperature (K)	100
*a*, *b*, *c* (Å)	10.0771 (3), 16.0292 (5), 17.8768 (6)
*V* (Å^3^)	2887.58 (16)
*Z*	8
Radiation type	Mo *K*α
μ (mm^−1^)	0.50
Crystal size (mm)	0.4 × 0.4 × 0.15

Data collection	
Diffractometer	Oxford Diffraction Xcalibur, Eos
Absorption correction	Multi-scan (*CrysAlis PRO*; Agilent, 2014 ▸)
*T*_min_, *T*_max_	0.954, 1.000
No. of measured, independent and observed [*I* > 2σ(*I*)] reflections	58593, 4475, 3679
*R*_int_	0.053
(sin θ/λ)_max_ (Å^−1^)	0.729

Refinement	
*R*[*F* ^2^ > 2σ(*F* ^2^)], *wR*(*F* ^2^), *S*	0.032, 0.077, 1.05
No. of reflections	4475
No. of parameters	174
H-atom treatment	H-atom parameters constrained
Δρ_max_, Δρ_min_ (e Å^−3^)	0.40, −0.33

Computer programs: *CrysAlis PRO* (Agilent, 2014 ▸), *SHELXS97* (Sheldrick, 2008 ▸), *SHELXL2018/3* (Sheldrick, 2015 ▸) and *XP* (Siemens, 1994 ▸).

## supplementary crystallographic information

### Crystal data

**Table:**

C_14_H_14_N_2_S_3_	*D*_x_ = 1.410 Mg m^−^^3^
*M**_r_* = 306.45	Mo *K*α radiation, λ = 0.71073 Å
Orthorhombic, *P**b**c**a*	Cell parameters from 10579 reflections
*a* = 10.0771 (3) Å	θ = 2.6–30.3°
*b* = 16.0292 (5) Å	µ = 0.50 mm^−^^1^
*c* = 17.8768 (6) Å	*T* = 100 K
*V* = 2887.58 (16) Å^3^	Tablet, pale yellow
*Z* = 8	0.4 × 0.4 × 0.15 mm
*F*(000) = 1280	

### Data collection

**Table:**

Oxford Diffraction Xcalibur, Eos diffractometer	4475 independent reflections
Radiation source: Enhance (Mo) X-ray Source	3679 reflections with *I* > 2σ(*I*)
Graphite monochromator	*R*_int_ = 0.053
Detector resolution: 16.1419 pixels mm^-1^	θ_max_ = 31.2°, θ_min_ = 2.3°
ω–scan	*h* = −14→14
Absorption correction: multi-scan (CrysAlisPro; Agilent, 2014)	*k* = −23→22
*T*_min_ = 0.954, *T*_max_ = 1.000	*l* = −25→25
58593 measured reflections	

### Refinement

**Table:**

Refinement on *F*^2^	Primary atom site location: structure-invariant direct methods
Least-squares matrix: full	Secondary atom site location: difference Fourier map
*R*[*F*^2^ > 2σ(*F*^2^)] = 0.032	Hydrogen site location: inferred from neighbouring sites
*wR*(*F*^2^) = 0.077	H-atom parameters constrained
*S* = 1.05	*w* = 1/[σ^2^(*F*_o_^2^) + (0.0318*P*)^2^ + 1.3474*P*] where *P* = (*F*_o_^2^ + 2*F*_c_^2^)/3
4475 reflections	(Δ/σ)_max_ = 0.002
174 parameters	Δρ_max_ = 0.40 e Å^−^^3^
0 restraints	Δρ_min_ = −0.32 e Å^−^^3^

### Special details

**Table:**

Geometry. All esds (except the esd in the dihedral angle between two l.s. planes) are estimated using the full covariance matrix. The cell esds are taken into account individually in the estimation of esds in distances, angles and torsion angles; correlations between esds in cell parameters are only used when they are defined by crystal symmetry. An approximate (isotropic) treatment of cell esds is used for estimating esds involving l.s. planes.

### Fractional atomic coordinates and isotropic or equivalent isotropic displacement parameters (Å^2^)

**Table:**

	*x*	*y*	*z*	*U*_iso_*/*U*_eq_	
S1	0.48621 (3)	0.63526 (2)	0.48864 (2)	0.01440 (8)	
C2	0.64877 (12)	0.62079 (7)	0.52085 (7)	0.0129 (2)	
N3	0.72242 (10)	0.57082 (6)	0.48059 (6)	0.0141 (2)	
C3A	0.65225 (12)	0.54010 (7)	0.42020 (7)	0.0135 (2)	
C4	0.70382 (14)	0.48489 (8)	0.36681 (7)	0.0169 (2)	
H4	0.793137	0.466208	0.369750	0.020*	
C5	0.62153 (14)	0.45833 (8)	0.30983 (7)	0.0194 (3)	
H5	0.654822	0.420983	0.273082	0.023*	
C6	0.48945 (14)	0.48568 (8)	0.30536 (7)	0.0198 (3)	
H6	0.435044	0.466538	0.265499	0.024*	
C7	0.43675 (14)	0.53990 (8)	0.35774 (7)	0.0179 (3)	
H7	0.347076	0.557869	0.354695	0.022*	
C7A	0.51995 (12)	0.56737 (7)	0.41532 (7)	0.0141 (2)	
C8	0.70646 (12)	0.65707 (7)	0.58882 (7)	0.0135 (2)	
C9	0.66224 (12)	0.72227 (7)	0.63140 (7)	0.0137 (2)	
C10	0.82871 (13)	0.61618 (8)	0.60980 (7)	0.0147 (2)	
N1	0.92273 (12)	0.58066 (7)	0.62713 (6)	0.0200 (2)	
S2	0.77453 (3)	0.76792 (2)	0.69408 (2)	0.01682 (8)	
C11	0.67428 (14)	0.80013 (8)	0.77350 (7)	0.0190 (3)	
H11A	0.723828	0.842357	0.802710	0.023*	
H11B	0.591943	0.826694	0.754915	0.023*	
C12	0.63786 (15)	0.72831 (9)	0.82435 (8)	0.0224 (3)	
H12A	0.584732	0.687626	0.796466	0.034*	
H12B	0.586321	0.749299	0.866859	0.034*	
H12C	0.718942	0.701444	0.842693	0.034*	
S3	0.50142 (3)	0.76155 (2)	0.62083 (2)	0.01684 (8)	
C13	0.53315 (14)	0.87150 (8)	0.60082 (8)	0.0192 (3)	
H13A	0.447342	0.900815	0.594773	0.023*	
H13B	0.579883	0.896822	0.643920	0.023*	
C14	0.61552 (15)	0.88404 (9)	0.53106 (8)	0.0242 (3)	
H14A	0.701489	0.856282	0.537151	0.036*	
H14B	0.629538	0.943837	0.522807	0.036*	
H14C	0.568927	0.860130	0.487976	0.036*	

### Atomic displacement parameters (Å^2^)

**Table:**

	*U*^11^	*U*^22^	*U*^33^	*U*^12^	*U*^13^	*U*^23^
S1	0.01247 (14)	0.01380 (15)	0.01694 (15)	0.00051 (11)	0.00032 (11)	−0.00103 (11)
C2	0.0125 (5)	0.0120 (5)	0.0143 (5)	−0.0005 (4)	0.0015 (4)	0.0019 (4)
N3	0.0150 (5)	0.0129 (5)	0.0143 (5)	−0.0007 (4)	0.0011 (4)	−0.0009 (4)
C3A	0.0158 (6)	0.0119 (5)	0.0129 (5)	−0.0019 (4)	0.0006 (4)	0.0020 (4)
C4	0.0197 (6)	0.0152 (6)	0.0158 (6)	0.0014 (5)	0.0009 (5)	−0.0003 (5)
C5	0.0268 (7)	0.0158 (6)	0.0157 (6)	0.0009 (5)	−0.0002 (5)	−0.0016 (5)
C6	0.0261 (7)	0.0171 (6)	0.0163 (6)	−0.0023 (5)	−0.0067 (5)	−0.0011 (5)
C7	0.0184 (6)	0.0153 (6)	0.0200 (6)	−0.0014 (5)	−0.0039 (5)	0.0021 (5)
C7A	0.0173 (6)	0.0106 (5)	0.0144 (5)	−0.0013 (4)	0.0003 (5)	0.0012 (4)
C8	0.0131 (6)	0.0127 (5)	0.0145 (5)	−0.0018 (4)	0.0018 (4)	0.0008 (4)
C9	0.0134 (6)	0.0127 (5)	0.0150 (6)	−0.0017 (4)	0.0024 (4)	0.0010 (4)
C10	0.0182 (6)	0.0137 (5)	0.0122 (5)	−0.0011 (5)	0.0010 (4)	−0.0028 (4)
N1	0.0218 (6)	0.0205 (5)	0.0177 (5)	0.0028 (5)	−0.0026 (4)	−0.0029 (4)
S2	0.01627 (15)	0.01690 (16)	0.01730 (15)	−0.00122 (12)	0.00140 (11)	−0.00523 (12)
C11	0.0250 (7)	0.0161 (6)	0.0159 (6)	0.0035 (5)	0.0025 (5)	−0.0049 (5)
C12	0.0231 (7)	0.0208 (7)	0.0232 (7)	0.0015 (5)	0.0051 (5)	0.0012 (5)
S3	0.01226 (15)	0.01522 (15)	0.02304 (17)	0.00007 (11)	0.00338 (11)	−0.00326 (12)
C13	0.0198 (6)	0.0139 (6)	0.0240 (7)	0.0024 (5)	−0.0015 (5)	−0.0002 (5)
C14	0.0270 (7)	0.0238 (7)	0.0218 (7)	−0.0025 (6)	−0.0011 (6)	0.0031 (5)

### Geometric parameters (Å, º)

**Table:**

S1—C7A	1.7371 (13)	C9—S3	1.7489 (13)
S1—C2	1.7519 (13)	C9—S2	1.7526 (13)
C2—N3	1.3078 (16)	C10—N1	1.1480 (17)
C2—C8	1.4672 (17)	S2—C11	1.8174 (13)
N3—C3A	1.3813 (16)	C11—C12	1.5121 (19)
C3A—C4	1.4015 (17)	C11—H11A	0.9900
C3A—C7A	1.4057 (17)	C11—H11B	0.9900
C4—C5	1.3807 (18)	C12—H12A	0.9800
C4—H4	0.9500	C12—H12B	0.9800
C5—C6	1.404 (2)	C12—H12C	0.9800
C5—H5	0.9500	S3—C13	1.8265 (14)
C6—C7	1.3835 (19)	C13—C14	1.512 (2)
C6—H6	0.9500	C13—H13A	0.9900
C7—C7A	1.3988 (18)	C13—H13B	0.9900
C7—H7	0.9500	C14—H14A	0.9800
C8—C9	1.3675 (17)	C14—H14B	0.9800
C8—C10	1.4449 (18)	C14—H14C	0.9800
			
C7A—S1—C2	88.97 (6)	S3—C9—S2	121.14 (7)
N3—C2—C8	118.28 (11)	N1—C10—C8	177.05 (14)
N3—C2—S1	115.52 (9)	C9—S2—C11	105.02 (6)
C8—C2—S1	126.18 (9)	C12—C11—S2	112.85 (9)
C2—N3—C3A	110.98 (11)	C12—C11—H11A	109.0
N3—C3A—C4	124.58 (12)	S2—C11—H11A	109.0
N3—C3A—C7A	115.03 (11)	C12—C11—H11B	109.0
C4—C3A—C7A	120.38 (12)	S2—C11—H11B	109.0
C5—C4—C3A	118.32 (12)	H11A—C11—H11B	107.8
C5—C4—H4	120.8	C11—C12—H12A	109.5
C3A—C4—H4	120.8	C11—C12—H12B	109.5
C4—C5—C6	121.01 (12)	H12A—C12—H12B	109.5
C4—C5—H5	119.5	C11—C12—H12C	109.5
C6—C5—H5	119.5	H12A—C12—H12C	109.5
C7—C6—C5	121.44 (12)	H12B—C12—H12C	109.5
C7—C6—H6	119.3	C9—S3—C13	101.91 (6)
C5—C6—H6	119.3	C14—C13—S3	112.70 (10)
C6—C7—C7A	117.76 (12)	C14—C13—H13A	109.1
C6—C7—H7	121.1	S3—C13—H13A	109.1
C7A—C7—H7	121.1	C14—C13—H13B	109.1
C7—C7A—C3A	121.08 (12)	S3—C13—H13B	109.1
C7—C7A—S1	129.43 (10)	H13A—C13—H13B	107.8
C3A—C7A—S1	109.49 (9)	C13—C14—H14A	109.5
C9—C8—C10	118.69 (11)	C13—C14—H14B	109.5
C9—C8—C2	129.40 (12)	H14A—C14—H14B	109.5
C10—C8—C2	111.90 (10)	C13—C14—H14C	109.5
C8—C9—S3	121.13 (10)	H14A—C14—H14C	109.5
C8—C9—S2	117.68 (10)	H14B—C14—H14C	109.5
			
C7A—S1—C2—N3	0.65 (10)	C2—S1—C7A—C7	178.99 (13)
C7A—S1—C2—C8	−177.35 (11)	C2—S1—C7A—C3A	−0.97 (9)
C8—C2—N3—C3A	178.07 (10)	N3—C2—C8—C9	165.41 (12)
S1—C2—N3—C3A	−0.10 (13)	S1—C2—C8—C9	−16.64 (19)
C2—N3—C3A—C4	−179.85 (12)	N3—C2—C8—C10	−13.23 (16)
C2—N3—C3A—C7A	−0.71 (15)	S1—C2—C8—C10	164.72 (9)
N3—C3A—C4—C5	179.09 (12)	C10—C8—C9—S3	−167.54 (9)
C7A—C3A—C4—C5	−0.01 (18)	C2—C8—C9—S3	13.90 (19)
C3A—C4—C5—C6	−0.11 (19)	C10—C8—C9—S2	15.10 (16)
C4—C5—C6—C7	−0.2 (2)	C2—C8—C9—S2	−163.46 (10)
C5—C6—C7—C7A	0.5 (2)	C8—C9—S2—C11	−146.43 (10)
C6—C7—C7A—C3A	−0.63 (19)	S3—C9—S2—C11	36.21 (9)
C6—C7—C7A—S1	179.41 (10)	C9—S2—C11—C12	77.62 (11)
N3—C3A—C7A—C7	−178.79 (11)	C8—C9—S3—C13	−123.70 (11)
C4—C3A—C7A—C7	0.39 (18)	S2—C9—S3—C13	53.57 (9)
N3—C3A—C7A—S1	1.17 (13)	C9—S3—C13—C14	59.99 (11)
C4—C3A—C7A—S1	−179.64 (10)		

### Hydrogen-bond geometry (Å, º)

**Table:**

*D*—H···*A*	*D*—H	H···*A*	*D*···*A*	*D*—H···*A*
C7—H7···S2^i^	0.95	3.02	3.6083 (13)	122
C12—H12*B*···S1^ii^	0.98	3.03	3.9677 (15)	161
C13—H13*A*···N3^i^	0.99	2.68	3.5746 (17)	151
C14—H14*A*···S1^iii^	0.98	2.91	3.7648 (15)	146
C14—H14*B*···N3^iv^	0.98	2.63	3.5277 (18)	152

Symmetry codes: (i) *x*−1/2, −*y*+3/2, −*z*+1; (ii) *x*, −*y*+3/2, *z*+1/2; (iii) *x*+1/2, −*y*+3/2, −*z*+1; (iv) −*x*+3/2, *y*+1/2, *z*.
